# Another Mechanism behind Cadmium Toxicity: Impaired NAT-Dependent Pathway Alters Chemical Biotransformation

**DOI:** 10.1289/ehp.118-a543a

**Published:** 2010-12

**Authors:** Rebecca Clay Haynes

**Affiliations:** **Rebecca Clay Haynes** has written for *EHP* since 1993. Her work has also appeared on National Public Radio and in the *Christian Science Monitor* and *The Environmental Forum*. In addition, she is the author of two children’s science books related to astronomy and space exploration

Cadmium is a complex carcinogen that may contribute to carcinogenesis through multiple mechanisms, including inhibition of enzymes that help repair damaged DNA. A new study provides information on another possible mechanistic pathway by revealing that cadmium impairs NAT-dependent carcinogen metabolism as demonstrated by altered biotransformation of environmental aromatic amines (AAs) **[*EHP*** **118(12):1685–1691; Ragunathan et al.]**.

Cadmium accumulates in body tissues and causes disease of the lungs, kidneys, liver, testes, prostate, and bladder. It also is thought to potentiate the carcinogenicity of many common workplace chemicals. NATs, or arylamine *N*-acetyltransferases, are metabolic enzymes known to play a major role in the biotransformation of exogenous chemicals including AAs, many of which are known or suspected human carcinogens.

In the current study the authors exposed purified NAT enzymes to cadmium and found the exposure led to rapid and irreversible functional impairment—removing the cadmium could not restore enzyme activities. They also exposed lung epithelial cells and laboratory mice to cadmium, then assessed NAT acetylation activity in the cells and mouse tissue samples. Biologically relevant concentrations of cadmium similar to those found in the lung tissue of heavy smokers impaired the NAT-dependent acetylation of carcinogenic AAs in lung epithelial cells, and NAT activity was strongly impaired in multiple tissues of mice exposed to cadmium.

These findings indicate acute cadmium exposure can alter the metabolism of carcinogenic AAs through impairment of the NAT-dependent pathway, with potentially important toxicologic effects—especially considering AAs are commonly found in cigarette smoke along with cadmium. The authors recommend further studies to address whether chronic cadmium exposure leads to similar effects.

